# Different longitudinal patterns of nucleic acid and serology testing results based on disease severity of COVID-19 patients

**DOI:** 10.1080/22221751.2020.1756699

**Published:** 2020-05-02

**Authors:** Zhang Yongchen, Han Shen, Xinning Wang, Xudong Shi, Yang Li, Jiawei Yan, Yuxin Chen, Bing Gu

**Affiliations:** a Department of Laboratory Medicine, The Second hospital of Nanjing, Nanjing University of Chinese Medicine, Nanjing, People’s Republic of China; b Department of Laboratory Medicine, Nanjing Drum Tower Hospital, Nanjing University Medical School, Nanjing, People’s Republic of China; c Department of Laboratory Medicine, Xuzhou Infectious Disease Hospital of Xuzhou Medical University, Xuzhou, People’s Republic of China; d Medical Technology School of Xuzhou Medical University, Xuzhou Key Laboratory of Laboratory Diagnostics, Xuzhou, People’s Republic of China; e Department of Laboratory Medicine, The Affiliated Hospital of Xuzhou Medical University, Xuzhou, People’s Republic of China

**Keywords:** COVID-19, SARS-CoV-2, serology testing, antibody responses, viral nucleic acid

## Abstract

Effective strategy to mitigate the ongoing pandemic of 2019 novel coronavirus (COVID-19) require a comprehensive understanding of humoral responses against severe acute respiratory syndrome coronavirus 2 (SARS-CoV-2), the emerging virus causing COVID-19. The dynamic profile of viral replication and shedding along with viral antigen specific antibody responses among COVID-19 patients started to be reported but there is no consensus on their patterns. Here, we conducted a serial investigation on 21 individuals infected with SARS-CoV-2 in two medical centres from Jiangsu Province, including 11 non-severe COVID-19 patients, and 5 severe COVID-19 patients and 5 asymptomatic carriers based on nucleic acid test and clinical symptoms. The longitudinal swab samples and sera were collected from these people for viral RNA testing and antibody responses, respectively. Our data revealed different pattern of seroconversion among these groups. All 11 non-severe COVID-19 patients and 5 severe COVID-19 patients were seroconverted during hospitalization or follow-up period, suggesting that serological testing is a complementary assay to nucleic acid test for those symptomatic COVID-19 patients. Of note, immediate antibody responses were identified among severe cases, compared to non-severe cases. On the other hand, only one were seroconverted for asymptomatic carriers. The SARS-CoV-2 specific antibody responses were well-maintained during the observation period. Such information is of immediate relevance and would assist COVID-19 clinical diagnosis, prognosis and vaccine design.

The ongoing outbreak of 2019 novel coronavirus (COVID-19), known as severe acute respiratory syndrome coronavirus 2 (SARS-CoV-2), was first reported in Wuhan, China in Dec 2019 [1]. As the outbreak of coronavirus disease 2019 (COVID-19) surges worldwide, this emerging pandemic has affected more than 1,200,000 patients globally. The dynamic profile of viral replication and shedding along with viral antigen specific antibody responses among COVID-19 patients started to be reported [2] but there is no consensus on their patterns. The longitudinal profiles of viral RNA and antibody response are urgently needed to guide clinical diagnosis, treatment, infection control and vaccine design [3].

In this respective study, we serially analysed the virus RNA test results in swab samples, along with anti-SARS-CoV-2 IgM and IgG responses among 21 COVID-19 patients at the Second Hospital of Nanjing and the Affiliated Hospital of Xuzhou Medical University in Jiangsu Province, China. Patients with suspected SARS-CoV-2 were confirmed after two sequential positive respiratory tract sample results. Throat swab samples were collected every 1–2 days. Anal swab samples were also obtained for RNA testing since 27 February 2020, as anal swab samples with prolonged viral shedding were observed during clinical practice [4]. Viral RNA was tested using real-time reverse transcriptional polymerase chain reaction (RT–PCR) kit (BGI Genomics, Beijing, China) as recommended by Chinese Center for Disease control and Prevention (CDC) following WHO guidelines [5]. The serum samples retrieved from routine biochemical or immunological testing were inactivated at 56°C for 30 min. These samples were later stored at −80°C for later serological detection. The IgG and IgM antibody responses against SARS-CoV-2 spike protein and nucleocapsid protein were tested by gold immunochromatography assay supplied by Innovita Co., LTd, China (CFDA approved).

The demographic information and disease severity of COVID-19 patients were obtained from their electronic medical records. Patients who had any of the following features during COVID-19 disease progression were classified as severe cases: (a) respiratory distress; (b) hypoxia (SpO_2_ ≤93%); (c) abnormal blood gas analysis (PaO2/FiO2 ≤ 300 mm Hg); or (d) severe disease complications including respiratory failure which requires mechanical ventilation, septic shock, or non-respiratory organ failure. The illness severity was defined according to the Chinese management guideline for COVID-19 (version 6.0) [6]. Asymptomatic carriers were defined as individuals who were positive for COVID-19 nucleic acid but without any symptoms during screening of close contacts. This study was approved by ethics committee of each medical centre, and information consent was waived as part of a public health outbreak investigation.

Between Jan 25 and March 18, 2020, 21 patients were enrolled including 11 (52.4%) non-severe COVID-19 patients, 5 (20.8%) severe patients, and 5 (20.8%) asymptomatic cases with SARS-CoV-2 infection. As of March 24, all patients have been clinical recovered and discharged. The characteristics of each group were summarized in Table 1. The dynamic viral shedding from throat swab and anal swabs were analysed (Figure 1). For non-severe patients, the respiratory swab remained positive for a median of 10 (range 2–21) days since symptom onset, whereas a median of 14 (range 9–33) days for severe patients. For asymptomatic cases, the period of positive respiratory swab lasted for a median of 18 (range 5–28) days. Despite of no statistical difference between groups, non-severe COVID-19 group was prone to become respiratory swab RNA negative in shorter period, compared to the group of severe patients and asymptomatic cases. Among 15 patients who tested for anal swab samples, 3 (18.75%) anal swabs remained positive for SARS-CoV-2 since their respiratory swab samples turned negative for SARS-CoV-2. Our serial SARS-CoV-2 RNA testing identified a prolonged viral shedding for asymptomatic cases compared to COVID-19 patients, suggesting the importance of early identification and timely quarantine for these asymptomatic carriers. Consistent with previous studies [7], we also found that the anal swab was able to maintain positive for weeks even after respiratory samples turned negative, validating the efficient replication in gastrointestinal tract during later stage of COVID-19 and a possible faecal-oral transmission route.

**Figure 1.**
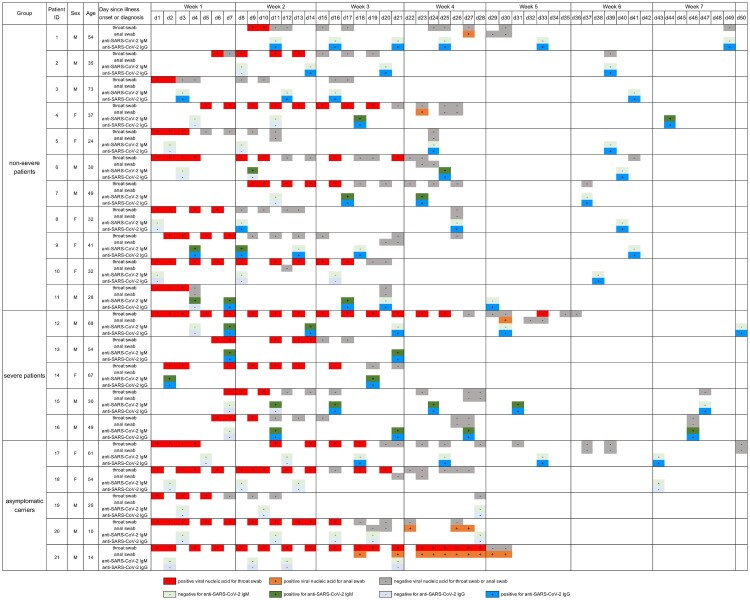
Detailed timeline of nucleic acid testing results for throat or anal samples along with the anti-SARS-CoV-2 IgM and IgG responses in 21 individuals infected with SARS-CoV-2, including 11 non-severe COVID-19 patients, 5 severe COVID-19 patients and 5 asymptomatic carriers. The timeline started from the symptom onset for both non-severe and severe COVID-19 patients, whereas the timelines started from the day of the diagnosis for asymptomatic carriers. F, female; M, male.

**Table 1.** Demographic characteristics, duration of viral RNA shedding and seroprevalence of SARS-CoV-2 infected individuals in our cohort.

	Non-severe cases (n = 11)	Severe cases (n = 5)	Asymptomatic carriers (n = 5)	Total (n = 21)
Age, years	35 (24–73)	54 (30–68)	25 (10–61)	37 (10–73)
Sex				
Female	5 (45.5%)	1 (20%)	2 (40%)	8 (38.1%)
Male	6 (54.5%)	4 (80%)	3 (60%)	13 (61.9%)
Duration of positive viral RNA in throat swab samples, day	10 (2–21)	14 (9–33)	18 (5–28)	14 (2–33)
Anti-SARS-CoV-2 seroprevalence	11 (100%)	5 (100%)	1 (20%)	17 (80.95%)

Data are presented as n (%) or median (range).

The longitudinal antibody responses were also determined in our cohort (Figure 1). All of 17 symptomatic patients (100%) were seropositive at the time point of discharge or during follow-up period. Specifically, among 11 non-severe patients, 3 (27.2%) patients seroconverted within week 1, 7 (63.6%) patients were anti-SARS-CoV-2 positive during week 2, while 9 (81.8%) patients showed positive antibody responses within week 3, and 11 (100%) patients were seropositive within week 6. For 8 (72.7%) of 11 patients, the first detection of antibody responses occurred during the period when their swab samples converted to RNA negative, suggesting that antibody responses might facilitate the viral clearance especially for non-severe COVID-19 patients. Furthermore, for all 5 severe COVID-19 patients, antibody responses were detected within 2 weeks. Of note, 3 out of 5 severe patients generated viral specific IgG responses prior to viral clearance. It is possible that significantly high level of SARS-CoV-2 viral load observed in severe cases [8,9] drives an early antibody response produced by immediate activation of extrafollicular B cells during acute infection [10,11]. Moreover, we also observed well-maintained antibody responses for all seroconverted individuals during our observation period, at least lasting for 6 weeks.

Only 1 (20%) out of 5 asymptomatic cases generated SARS-CoV-2 specific antibody responses (Table 1 and Figure 1), and this patient (patient 17) was not seroconverted until week 3 since her diagnosis. Consistent with her delayed antibody responses, the throat swab sample converted to RNA negative as late as week 3. For the remaining 4 asymptomatic patients, 2 patients were not seroconverted within week 2 and week 3, respectively; while 2 patients remained anti-SARS-CoV-2 negative during week 4. It is not known whether they become seropositive later. It is possible that such seronegative asymptomatic carriers were resulted from low level of viral load, but the false positive nucleic acid test results cannot be ruled out.

Our current study has revealed important implications to understand the dynamic interplay between SARS-CoV-2 and humoral responses. First, seroconversion was observed in 100% (17/17) of symptomatic patients during the observation period, suggesting that the serological test could serve as a complementary testing assay to nucleic acid test for those symptomatic COVID-19 patients, especially given the potential false negative viral RNA results using throat swab samples. Nevertheless, we also noted only one person with humoral responses among asymptomatic carriers. Additional viral and immunological analyses are warranted to understand this observation. Better understanding on the biological significance of asymptomatic cases without seroconversion is urgently needed.

Although it is generally considered a beneficial role of specific antibody response during viral infection, we did not identify a strong association of seroconversion and disease severity in our cohort. For both non-severe and severe COVID-19 cases, the viral specific antibody responses were detected. Meanwhile, our study revealed an early induction of antibody responses in severe cases. Consistently, a recent study also revealed that high level of antibody titer might be independently associated with a worse disease severity for COVID-19 patients [12]. A similar clinical observation was also observed from a previous study of severe acute respiratory syndrome coronavirus (SARS-CoV), in which deceased infected patients reached the peak of anti-spike neutralizing antibody much earlier than that of the clinical recovered patients [13]. However, only measurement of peripheral antibody responses may not be sufficient. We can also speculate that high level of initial viral load early in infection may lead to severe COVID-19 cases. Such high viral load can drive strong extrafollicular B cell responses leading to rapid antibody responses which do not follow the sequence of IgM to IgG development stages [10,11]. Such high quantity of antibodies produced from extrafollicular B cells can contribute greatly to the inflammatory responses by promoting monocyte and macrophage accumulation and the massive cytokine storm including IL-8 and MCP-1, which might be responsible for fatal acute lung injury, as indicated during SARS-CoV infection [14]. Also, antibody might facilitate the infection of target cells by promoting the uptake of virion-antibody complex via Fc receptors (FcR) [15]. Whether the acute anti-SARS-CoV-2 antibody responses attributed to disease progression of COVID-19 disease deserves further investigation. On the other hand, a graduate development of viral antigen specific B cells undergoes the somatic hypermutation and affinity maturation at the traditional germinal centre, which ultimately lead to high affinity protective antibody responses as observed in non-severe COVID-19 patients.

Our study also has several limitations. First, our study is limited by small sample size, thereby it is not known whether our finding could be generalized. Nevertheless, our cohort is representative of different COVID-19 disease spectrum, including non-severe cases, severe cases, and asymptomatic carriers. Furthermore, the quantitative viral load and titers of antibody response were not available, so the kinetics of viral shedding and the magnitude of antibody response during COVID-19 disease progression remained unknown in this study.

Collectively, our study of serial nucleic acid and serological testing among various COVID-19 patients indicated distinct dynamic patterns among three groups of SARS-CoV-2 infected individuals. Our findings contribute to the evolving understanding of the sophisticated interaction between this emerging SARS-CoV-2 virus and host immune system.
